# Preparation and Characterization of Eco-Friendly Transparent Antibacterial Starch/Polyvinyl Alcohol Materials for Use as Wound-Dressing

**DOI:** 10.3390/mi13060960

**Published:** 2022-06-17

**Authors:** Mohammad Mohsen Delavari, Ion Stiharu

**Affiliations:** Department of Mechanical, Industrial and Aerospace Engineering, Concordia University, Montreal, QC H3G 1M8, Canada; ion.stiharu@concordia.ca

**Keywords:** starch-based films, transparent, polyvinyl alcohol, ultrasound mixing, wound-dressing, characterization, surface morphology

## Abstract

In this study, eco-friendly and transparent starch-based/polyvinyl alcohol/citric acid composite films are evaluated for their efficacy as wound dressing materials. The starch/polyvinyl alcohol (PVA) materials with added citric acid (0.46–1.83 wt%) and glycerol were made and handled based on the modified casting method. This new formulation decreases the amount of PVA used in the conventional preparation method. Citric acid ensures an appropriate antibacterial environment for wound-dressing materials. The mechanical, chemical, and surface morphological properties of such films were assessed and analyzed by tensile strength tests, UV–Vis spectrometry, swelling index, and scanning electron microscopy (SEM). Furthermore, the water vapor transmission (WVT) quantity was measured for an ideal wound-healing process to investigate an optimal moisture environment around the wound bed. Moreover, the pH level of the dressings was measured to examine the possibility of bacterial growth around these starch-based films. Additionally, the films’ in-vitro antibacterial activities were studied against the two most common Gram-positive and Gram-negative bacteria (Escherichia coli and Staphylococcus aureus). The new starch-based dressings demonstrated suitable degradation, antibacterial activity, fluid absorption, and adequate mechanical strength, representing wound-dressing materials’ vital features.

## 1. Introduction

The skin is the largest organ of the human body, but it is among the least known ones. This is because its function is more schematically defined and measured than the other systems in the body. It is widely recognized that chronic wounds are a serious public health problem. Developing pharmaceutical dosage forms to ensure patient comfort and safety and optimize treatment effectiveness are among the most essential biomedical, pharmaceutical, and biomaterial research areas [1]. Based on a recent study conducted by the World Health Organization (WHO), skin burns globally represent one of the leading causes of death and disability. Most fatalities occur in underdeveloped regions, two-thirds in African and Asian nations [2]. Immobility, physical limitations, continuous pain, and psychological trauma are the most common effects of chronic wounds that can negatively affect injury and mortality rates, temporarily or permanently [3].

Wound-healing occurs in four phases: homeostasis, inflammatory response, proliferation, and remodeling [4]. Research has now shown that a wound requires a moist environment that emulates the functions of the skin [5]. The length of time involved and the type of dressing used vary and interfere with the healing process in chronic skin ulcer treatment. During conventional treatment methods, the steps of removing bandages, cleaning the wound, administering medicines, and replacing new covers on the wound are repeated, sometimes more than once a day, which causes significant pain and discomfort to the patient and delays wound-healing [6,7].

The development of new transparent natural polymeric wound dressings has various advantages over conventional ones, such as drug delivery systems, biodegradability, low price, physical protection, and antibacterial activity around the wound area [8]. Moreover, these ideal dressings should provide a reasonable water vapor transmission rate (WVTR), absorption of the wound exudates, and from a mechanical point of view, high tensile strength and flexibility are essential characteristics [9].

Furthermore, due to recent environmental concerns reflected by global warming, many researchers are focused on developing natural and synthetic polymer-based materials [10]. Since conventional petroleum-derived materials have excellent mechanical properties and a straightforward fabrication process, they aid in the extensive implementation of these plastics (polystyrene, polyvinyl chloride, and nylon) in different biomedical and packaging industries and agricultural applications [10,11]. Substituting plastics with biodegradable polymers, such as starch and polyvinyl alcohol (PVA), for example, represents a considerable breakthrough in tackling those concerns. Recently, Starch/PVA-based materials for general and functional applications have been implemented in food packaging [11,12], wound-dressing [5,13,14,15], wound-healing [6], and drug delivery systems [16].

PVA, a water-soluble, odorless, and non-toxic polymer [17] produced from polyvinyl acetate through hydrolysis, is a polymer extensively used in industrial as well as biomedical fields. Researchers have recently recognized PVA as a viable alternative to synthetic polymers because of its biodegradability and outstanding mechanical properties [18,19]. Additionally, PVA possesses functionalities, such as emulsifying, adhesive, and film-forming properties, that make it a promising candidate for use in wound dressings [20]. In general, these favorable properties are related to the presence of OH groups and the ability to form hydrogen bonds via electrostatic forces [21].

However, PVA is relatively expensive and exhibits oxygen barrier properties; therefore, to eliminate these flaws, pure PVA could be mixed with starch, known as a natural polymer [22,23]. Many research teams have used renewable, abundant, and natural materials, such as starch, to prepare biodegradable starch/PVA-based blends [12,14,18]. Starch-based films are transparent, biocompatible, non-toxic, and, more importantly, they can naturally degrade in a short time-period in water or soil [24]. Starch is vital in forming a continuous matrix in the material solution [25]. Starch and PVA are incompatible with one another, and therefore, there have been many proposed techniques to strengthen starch blend characteristics, and the focus has been on the compatibility between starch and PVA [26].

Citric acid (CA) can be used as a crosslinking agent, considered non-toxic in the solution [27,28,29]. It has been reported that citric acid improved the films’ flexibility and created an antibacterial environment on and around the starch/PVA film by increasing the CA percentage content [29,30]. Moreover, due to the suitable acidity and the Food and Drug Administration (FDA) approval as a toxically safe organic acid, citric acid in appropriate proportions should enhance the antibacterial characteristic of the wound-dressing films to promote the efficacy of new materials in the wound-healing process [14,15,31].

Moreover, the various concentrations of citric acid or glycerol were considered additives for starch/PVA blends by Yoon et al. [32]. This team investigated the effect of those additives on the degree of swelling, ultimate tensile strength (UTS), elongation, and solubility. It was concluded from their research that multiple carboxyl groups in the citric acid facilitated better properties except for the degree of swelling than those obtained with glycerol [32]. Despite this, their study does not yield definitive conclusions regarding the applicability of these films as wound dressings. In addition, Yoon et al. did not consider the optimality of the PVA/St/CA film components and the process parameters during fabrication at the lower crosslinking temperature. Shi et al. [31], Wu et al. [28], and Das et al. [14,15] reported that for starch/PVA/GLY/CA wound-dressing films, a reduction in the degree of swelling was noticed by increasing CA concentration in solution while the elongation-at-break percentage consistently increased. Therefore, CA could be utilized to enhance the flexibility characteristics of PVA/starch blends. However, the ingredient percentages, such as the PVA and starch weight, used in their film preparation steps were high, considering the amount of DI water (solvent) used in their formulation. This led to a much longer preparation and drying duration using higher oven temperatures. Birck et al. fabricated a PVA/CA film without considering the impact of starch on the properties of PVA composite film. In addition, the authors did not optimize the crosslinking temperature and used high-temperature processing. Furthermore, parameters related to characterization have not been extensively investigated [33]. Studies of degradation, water vapor transmission rate, and antibacterial effectiveness were not considered by Shi et al. [31].

In light of the gaps identified in the literature, this study evaluates the optimality of the solution casting process during the fabrication of starch-based/PVA films at much lower casting and drying temperature than the most relevant literature and utilizes the ultrasound mixer in the preparation process. Another goal of the carried-out investigation was to observe the compatibility of starch-based/PVA/citric acid films as an ideal wound dressing material. When evaluating the efficacy of starch-based films as wound dressing materials, all pertinent characterization factors, including swelling index, in vitro degradation, mechanical strength, antibacterial activity, and water vapor transmission rate were considered.

## 2. Materials and Methods

### 2.1. Materials

Sigma Aldrich, (Oakville, ON, Canada), provided the polyvinyl alcohol (M_w_ 20,000–23,000 g/mol and 88% hydrolyzed), potato starch (M_w_ 342.30 g/mol), and citric acid (M_w_ 192.12 g/mol). The glycerol (M = 92.05 g/mol; purity = 99.0%) was purchased from Fisher Scientific. The deionized water (DI) was collected from a standard setup. The following materials were purchased from Sigma Aldrich, (Burlington, MA, USA): Penicillin G sodium salt (Sigma 13752), *Staphylococcus aureus* (*S. aureus* S2014), and *Escherichia coli* (*E. coli* EC1).

### 2.2. Preparation of the Films

Initially, DI water was added to the PVA, and the mixture was stirred for 15 min at 300 rpm under a temperature of 80 °C. In the meantime, an ultrasonic device was used to mix the starch and DI water in order to produce a homogeneous solution. After that, both solutions were mixed and stirred for 15 min until a transparent viscous solution was achieved. The solution was supplemented with glycerol and citric acid in 10-min increments for each component. Once the bubbles were removed from the solution, the blended solution was poured onto the flat glass at room temperature. The stirring speed and temperature of the solution were maintained throughout the preparation process. After numerous observations, several experiments were conducted to optimize the drying temperatures and drying time so that a complete drying process would occur without any extra moisture being left in the film, which would result in a thinner film with improved stretching and transparency and no signs of brittleness. This step begans with 12 h of drying in ambient conditions, and then the blend was heat-treated for 30 min at 95 degrees Celsius. Finally, the starch/PVA blend was slowly removed from the glass within the next two hours (Figure S1). In order to prevent contamination, the films were then stored inside airtight covers. Table 1 details the composition of blended substances.

### 2.3. Characterizations

#### 2.3.1. Film Thickness

This test was conducted using a digital caliper (DC-4150, Shanghai, China), which provided an accuracy of 0.001 mm when measuring the dried thickness of the starch/PVA/CA/GLY films. The ultimate tensile strength of the film was calculated by taking five thickness measurements along the gauge length of each specimen. The results are shown in Figure S2.

#### 2.3.2. Swelling Index

For the purpose of evaluating the fluid absorption capacity of these films, it is necessary to apply the swelling index methodology. As part of this method, samples (1 × 1 cm^2^) were immersed in phosphate buffer saline (PBS) solution and incubated at 37 °C for 24 h [14]. The swelling index (%) is calculated by utilizing Equation (1), where *W*_0_ and *W_s_* are, respectively, the dry weight and wet weight after immersion in a PBS solution.

(1) $$S.I.=\frac{W_{s}}{W_{0}}\times 100\%$$

#### 2.3.3. Weight Loss

The speed and efficiency of wound-healing are influenced by a number of factors, one of which is the degradation of dressings. It is through this process that drug molecules can be released into the wound area in order to prevent infections. Using in vitro degradation methods, it was possible to evaluate how much weight was lost after immersion in PBS solution with 7.4 pH at 37 °C for 14 days. Equation (2) can be used to determine the weight loss percentage value [14]; *W*_0_ is the dry weight of the samples before immersion in the PBS media or saline and *W_f_* is the weight of the pieces after drying at 37 °C.

(2) $$\mathrm{WL}\%=\frac{W_{0}-W_{f}}{W_{0}}\times 100\%$$

#### 2.3.4. Mechanical Strength

In order to ensure prepared films are durable and flexible enough to be used as wound dressings and wearable electronics, we developed a high-resolution testing device [34]. The ASTM D882–10 test was conducted in ambient conditions (25 °C, 25% relative humidity). According to the standard, the dumbbell-shaped films were mounted in two jaw grips, and the motor speed was set at 50 mm/min. Five samples were tested for ultimate tensile strength and elongation-at-break; five values were used to calculate the average.

#### 2.3.5. Water Vapor Transmission Measurement

Wound-healing is dependent on a set of environmental parameters, such as moisture and oxygen [35]. As a result, the water vapor transmission rate (WVTR) was determined by implementing the standard ASTM E398 recommendations. As part of these tests, 60 mm diameter samples were kept under 25% relative humidity (RH) and 25 °C temperatures for 24 h to reach equilibrium.

#### 2.3.6. Antibacterial Activity and pH Levels

We evaluated the antibacterial properties of starch-based materials using two of the most relevant Gram-positive and Gram-negative bacterial pathogens. In this test, bacteria, such as *Staphylococcus aureus* and *Escherichia coli*, were prepared, and the inhibition zones of each sample were measured utilizing ImageJ (1.53 k; National Institutes of Health, Bethesda, MA, USA) open-source software. Bacteria were grown on petri dishes over agar media in a bacterium culture incubator at 37 °C with 5% CO_2_. In order to measure the pH levels of the samples, a calibrated OMEGA waterproof pH tester, model PHH-7011 [34], was used for this purpose.

#### 2.3.7. UV–Vis Spectroscopy

Following our previous study [34], samples (S1-S4) were cut into appropriate shapes and positioned in a UV–Vis spectrophotometer (LABOMED INC. UV-2550) following the dressing light transmission validation. Light transmission properties of film samples (S1–S4) measured from 250 nm to 1000 nm.

#### 2.3.8. The Scanning Electron Microscopy (SEM)

At a voltage of 15 kV, the films (S1–S4) were examined by scanning electron microscopy (SEM, Hitachi S 3400N). SEM with energy-dispersive X-ray spectroscopy (EDX) was used to analyze the dressings (S1–S4). Each of the film samples was adhered to a 1.5 × 1.5 cm^2^ piece of carbon tape that was attached to the metallic stubs. The samples’ images of the distribution of the clustered starch in granules on the materials’ surface were processed using the ImageJ software (1.53 k; National Institutes of Health, Bethesda, MA, USA).

## 3. Results

### 3.1. Mechanical Strength of PVA/Starch Blend Films

**Figure 1:**
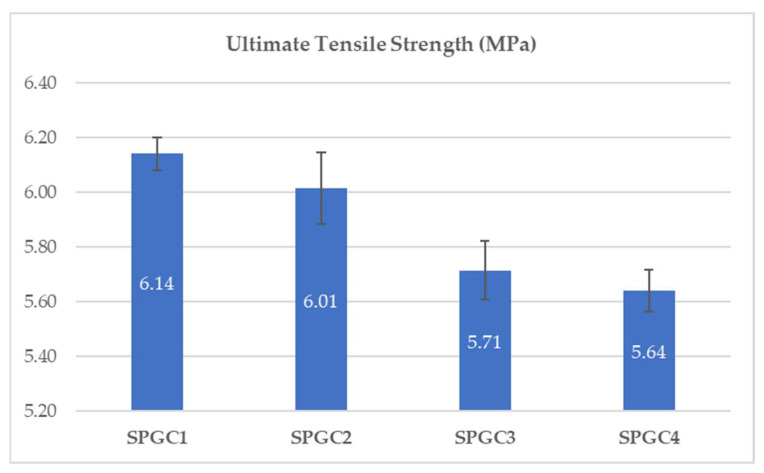
The effects of citric acid on the mechanical properties of biodegradable starch-based films [citric acid concentrations (0.46, 0.92, 1.38, and 1.83 wt%)].

The composite films need to have appropriate mechanical properties in order to provide an individual with physical protection for wounds. In order to determine the tensile strength of the starch-based/PVA matrix in response to increasing citric acid (CA) concentrations, a series of experiments was carried out. As shown in Figure 1, the films’ ultimate tensile strength ranged between 5.64–6.14 MPa, in spite of the slight increase in the amount of CA in the solution. CA was found to not only affect plasticization but also induce crosslinking within the polymer matrix during this experiment. In this paper, since the thicknesses of the samples used in determining the ultimate tensile strength are relatively close, the effect of the samples’ thicknesses on the dressings’ ultimate tensile strength is negligible.

A measure of flexibility or percentage of elongation-at-break relates to the extendibility of film from the initial length up to where the film breaks. Elongation is able to withstand bending of the film shape during handling, allowing for a shorter time of wound-healing. As a result, ensuring elongation strength characteristics is another critical property for wound dressings.

**Figure 2:**
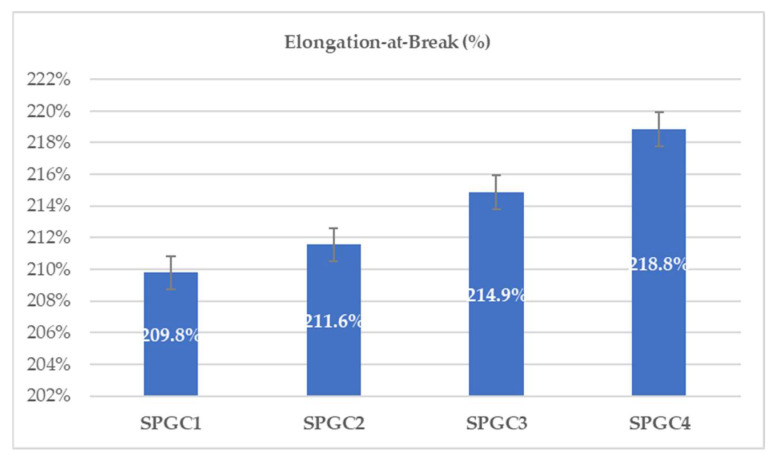
The effects of various citric acid proportions (0.46, 0.92, 1.38, and 1.83 wt%) on the film elongation-at-break percentage.

As the solution’s CA percentage increased from 0.46 wt% to 1.83 wt%, the elongation-at-break increased by 9% (Figure 2). After adding CA to the film solution, the CA residual increased in blends, which reduced the macromolecule interactions [31] and led to a decrease in UTS (Figure 1) of the films and an increase in the elongation-at-break as a result of CA’s plasticization property (Figure 2). Adding citric acid to the solution led to a marked decrease in Young’s modulus (Table 2). Young’s moduli ranged from 25.7 to 29.2 MPa. 0.46 weight percent citric acid in the polymer blend gave the best properties (TS of 6.14 MPa and Young’s modulus of 29.2 MPa). Citric acid crosslinks PVA and starch molecules, generating –C=C– groups at the expense of –OH groups.

### 3.2. Swelling Index of Starch-Based Blend Films

**Figure 3:**
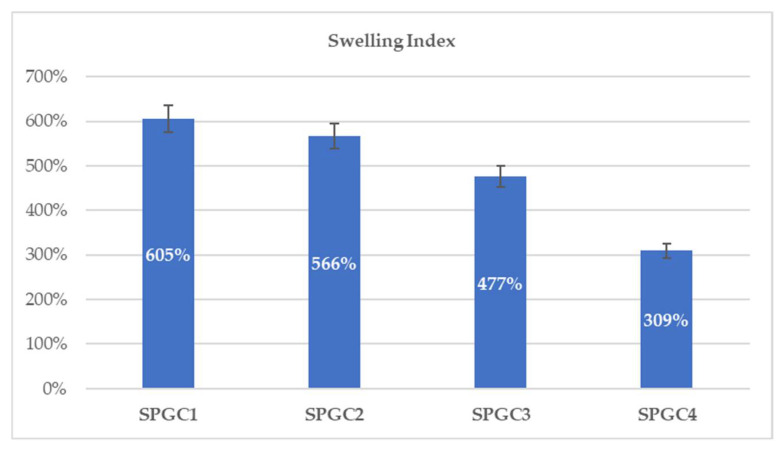
The effects of different citric acid concentrations on starch-based materials swelling index.

Figure 3 shows the effect of changing the concentration of citric acid in the solution on the swelling index. The swelling index decreases from 605 to 309 percent when the mixing temperature is maintained at 80 °C, and the citric acid concentration varies between 0.46 and 1.83 wt%. Increases in citric acid concentrations result in a decrease in swelling indexes as a consequence. The higher citric acid concentration and crosslinking temperature promote significantly higher crosslinking between starch and PVA granules, which reduces the swelling index. Wound-dressing materials with a high swelling index can effectively absorb wound exudates. Starch-based films that are crosslinked at temperatures of 80 °C are able to have a swelling index of at least 300 percent when tested at different citric acid concentrations. In addition, in light of the fact that greater crosslinking promotes greater mechanical strength (as shown in Figure 1), starch-based films tend to have another advantage in this regard.

### 3.3. In Vitro Degradation of Starch-Based Blends in PBS

**Figure 4:**
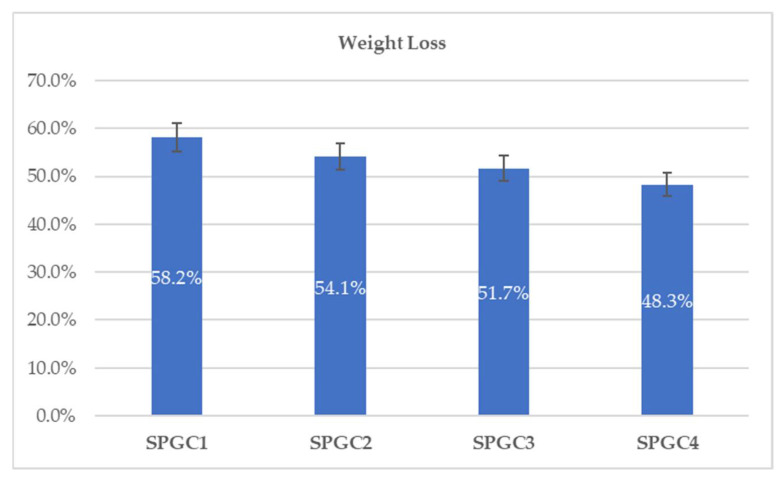
The weight loss percentage for starch-based wound-dressing films.

A weight-loss study of starch-based/citric acid films (SPGC1-SPGC4) was conducted after 14 days of degradation in phosphate-buffered saline (7.4 pH). Compared to one another, samples’ weight loss was reduced slightly, and the weight loss value for SPGC1, SPGC2, SPGC3, and SPGC4 samples was recorded at 58.2%, 54.1%, 51.7%, and 48.3%, respectively (Figure 4). During the degradation of polymers, water plays an integral role by accelerating enzyme diffusion into polymer chains and resulting in chain cleavage [36]. An ester bond in a film provides resistance to moisture (water) penetration [29] and an increase in hydrophobicity. Consequently, the degradation of starch/PVA membranes is mainly caused by the breakdown of crosslinking segments created by CA between the PVA and starch molecular structures. As a result, the biodegradation rate decreased by increasing CA percentages in the formulation of the materials. This trend is consistent with those reported in the related literature [14,15].

### 3.4. Water Vapor Transmission Rate of Starch-Based/PVA Films

**Figure 5:**
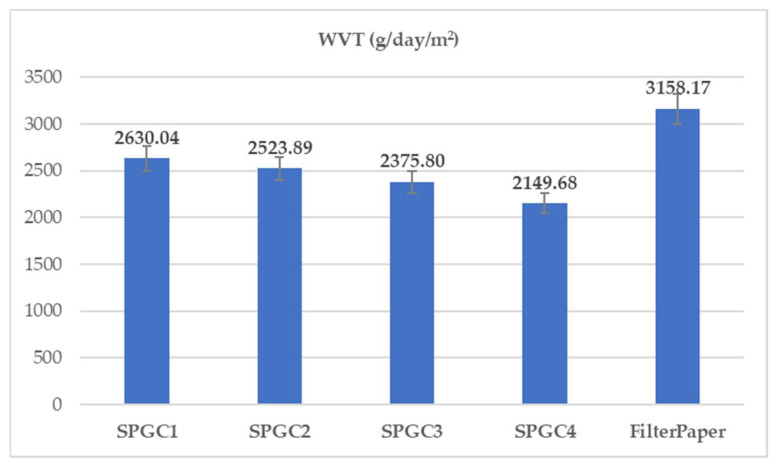
Water vapor transmission rate for starch-based wound-dressing films.

An optimal water vapor transmission (WVT) rate of 2000–2600 g/m^2^/day is crucial to avoiding dryness of the wound due to the fact that low humidity and the accumulation of exudates in the wound area might lead to bacterial infection [37]. On the other hand, the type of skin wound defines the WVT rate; in other words, the first-degree burns and the granulating wounds need the WVT of 300 and 5000 g/m^2^/day, respectively [37,38]. The starch-based/PVA/CA blends (SPGC1-SPGC4) WVT were assessed from 2630.04 g/m^2^/day to 2149.68 g/m^2^/day (Figure 5). Compared to a relatively porous material, such as filter paper, the starch-based materials revealed adequate water vapor transition rates implemented in wound dressings to provide enough humidity to have the best wound-healing steps.

### 3.5. Antibacterial Effectiveness and pH Levels of Starch-Based Films

The antibacterial activity test is a vital component of the wound dressing. In addition to its acidity and FDA approval as a non-toxic substance, citric acid is a promising organic acid. When used in an appropriate proportion, citric acid should not inhibit cell growth but also contribute to the antibacterial properties of the wound dressing film to promote the growth of new tissues during wound-healing [14].

**Figure 6:**
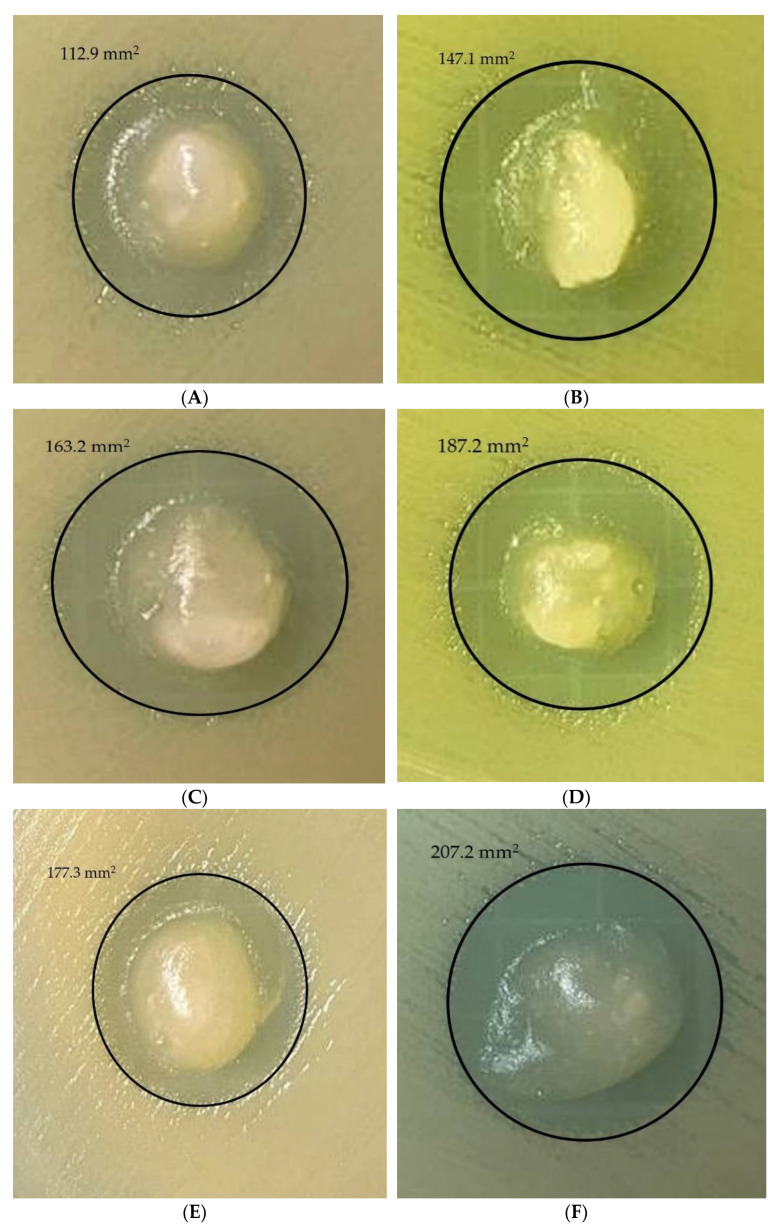

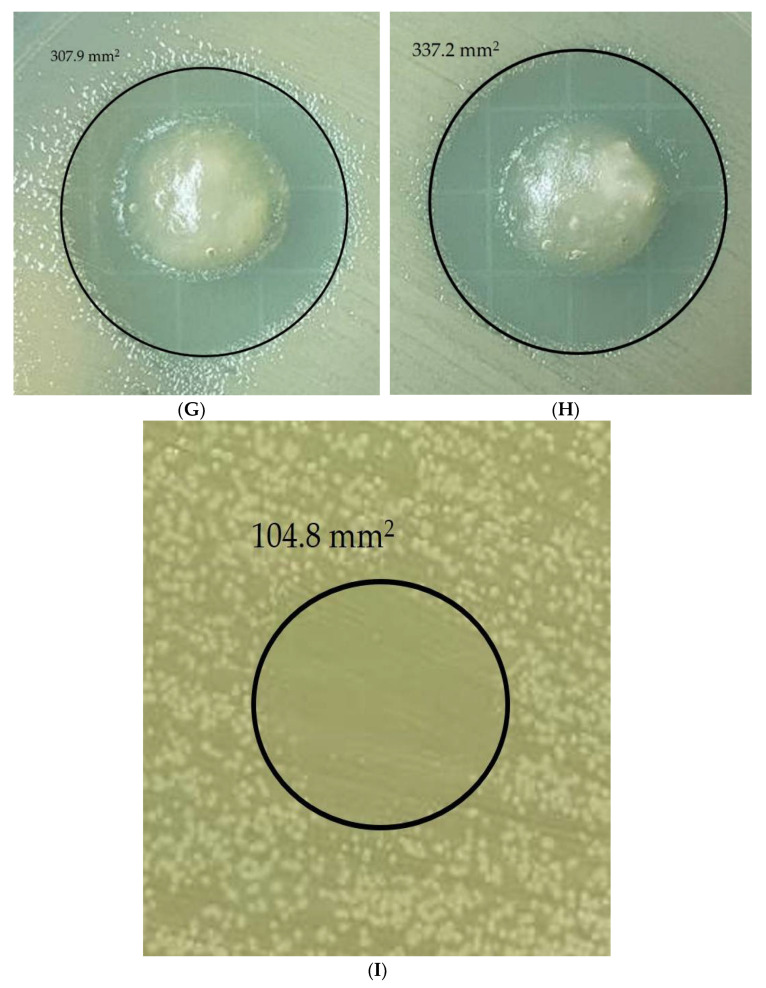
A study of the antibacterial activity of different formulations of starch-based wound-dressings against *E. coli* (**A**) 0.46 wt% CA, (**B**) 0.92 wt% CA, (**C**) 1.38 wt% CA, (**D**) 1.83 wt% CA and against *S. aureus* (**E**) SPGC1, (**F**) SPGC2, (**G**) SPGC3, (**H**) SPGC4), (**I**) control zone.

In vitro tests were conducted using starch-based films and penicillin as a control (Figure 6I) against Staphylococcus aureus (Gram-positive bacteria) and Escherichia coli (Gram-negative bacteria). According to Figure 6, all starch/PVA/CA films showed negative bacterial growth around the film surface area by creating a clear, circular inhibition zone against *E. coli* (Figure 6A–D) and *S. aureus* (Figure 6E–H). A high level of antibacterial efficacy was demonstrated by the starch-based dressings prepared with 1.38 wt% and 1.83 wt% of CA among all samples tested. The starch-based composite film with a CA concentration of 1.83 wt% was found to be the most effective against *E. coli* and *S. aureus*, with inhibition zones of 187.2 mm^2^ (Figure 6D) and 337.2 mm^2^ (Figure 6H), respectively.

CA’s antibacterial properties are due to its acidic nature, which facilitates the breakdown of bacterial protein and cell membrane synthesis. As a result, CA is a reputed organic acid that is suitable as a low-cost additive to further optimize the antibacterial properties of wound-dressing starch-based/PVA composite films.

The existing literature indicates that the pH levels of the skin under normal circumstances are between 4 and 6, which is insufficient to prevent the growth of bacteria [39]. As an example, human skin contains a substantial amount of *Staphylococcus Aureus*, which may worsen an eczema patient’s condition. The pH values between 7.5 and 8.9 are likely to aid the reproduction of these bacteria [40]. In this sense, an acidic environment will play a critical role in helping to adjust the antibacterial activities in the wound bed.

At 37 °C, the pH levels of the starch-based solutions (SPGC1-SPGC4) were 3.94, 4.12, 4.25, and 4.43, respectively, as measured by the Omega pH tester model PHH-7011. Therefore, these materials can offer an appropriate pH level that inhibits the growth of bacteria around the wound milieu after contacting the internal body fluids.

### 3.6. Ultraviolet–Visible Spectroscopy

**Figure 7:**
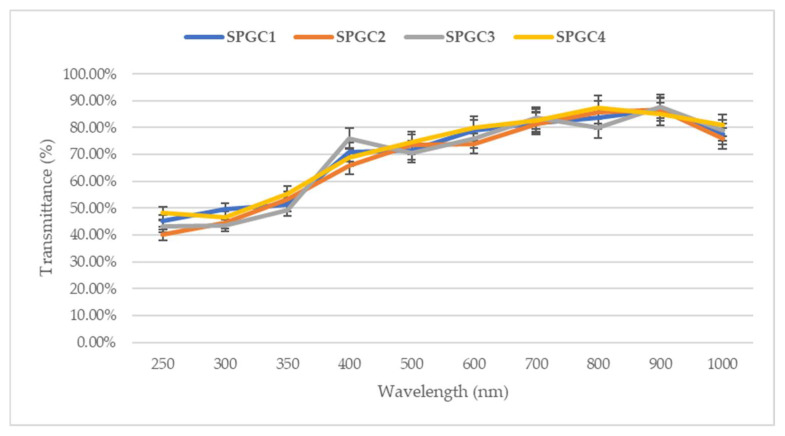
UV–Vis transmittance percentage of starch-based/PVA wound dressings (SPGC1–SPGC4).

The reflective properties of the starch-based/PVA samples (SPGC1-SPGC4) were measured to observe the materials’ light absorption (Figure 7). At a lower wavelength, the transmittance average percentage is around 45% and increasing. What stands out in Figure 7 is a sharp rise in the transmittance value at 400 nm wavelength, which could be due to the complexation between polymers, plasticizer (citric acid), and crosslinking agent (glycerol). Additionally, the samples (SPGC1-SPGC4) showed the same response based on the fact that these materials are transparent and have all the same polymer structures.

### 3.7. SEM

**Figure 8:**
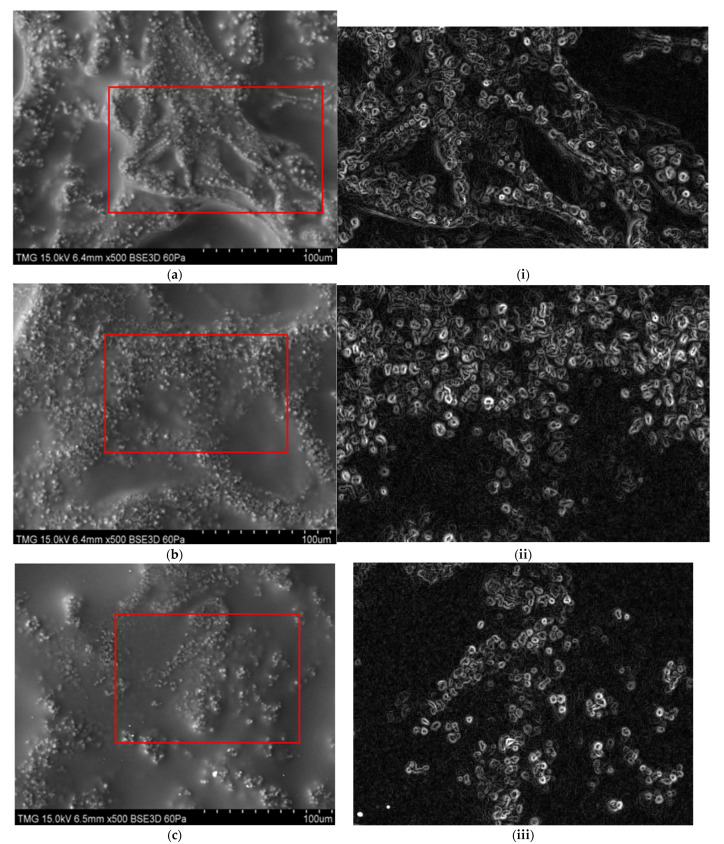

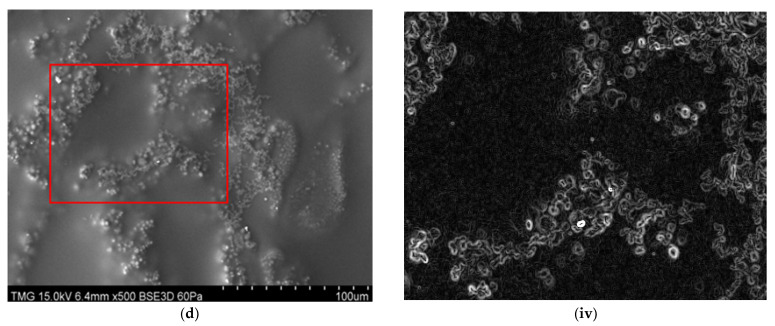
SEM images of starch-based/polyvinyl alcohol/citric acid/glycerol (SPGC) films, (**a**) 0.46 wt% CA (SPGC1), (**b**) 0.92 wt% CA (SPGC2), (**c**) 1.38 wt% CA (SPGC3), (**d**) 1.83 wt% CA (SPGC4); The analyzed ImageJ software images without changing the magnification of SEM images; (**i**) 0.46 wt% CA (SPGC1), (**ii**) 0.92 wt% CA (SPGC2), (**iii**) 1.38 wt% CA (SPGC3), (**iv**) 1.83 wt% CA (SPGC4).

The SEM photographs that are commonly used to study surface morphology and density of composite films have been used to analyze a wide variety of composite films. Taking into account the upper surface images, it has been determined that increasing citric acid contents from 0.46 wt% to 1.83 wt% in the starch-based/PVA composite films did not significantly alter their surface morphology (Figure 8a–d). A partial section of each SEM image, without changing the magnification, was processed and analyzed by ImageJ software (Figure 8i–iv), indicating that citric acid may help distribute, interconnect, and integrate throughout the polymer matrix of the films. Crosslinked polymer chains formed a densely packed structure due to their crosslinked networks (Figure 8i–iv). Each of the images (i–iv) in Figure 8 was analyzed utilizing the ‘find maxima’, ‘find edges’, and ‘enhanced contrast’ functions of the ImageJ software (8-bit; 96 K; 1.53 k; National Institutes of Health, Bethesda, MA, USA).

**Figure 9:**
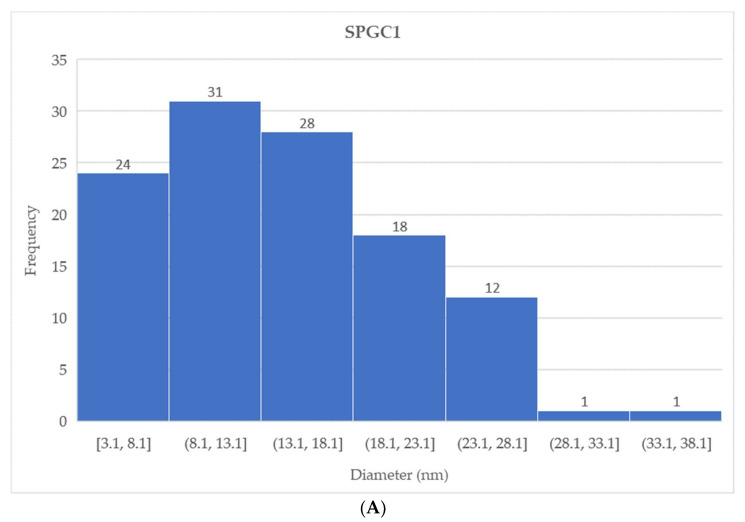

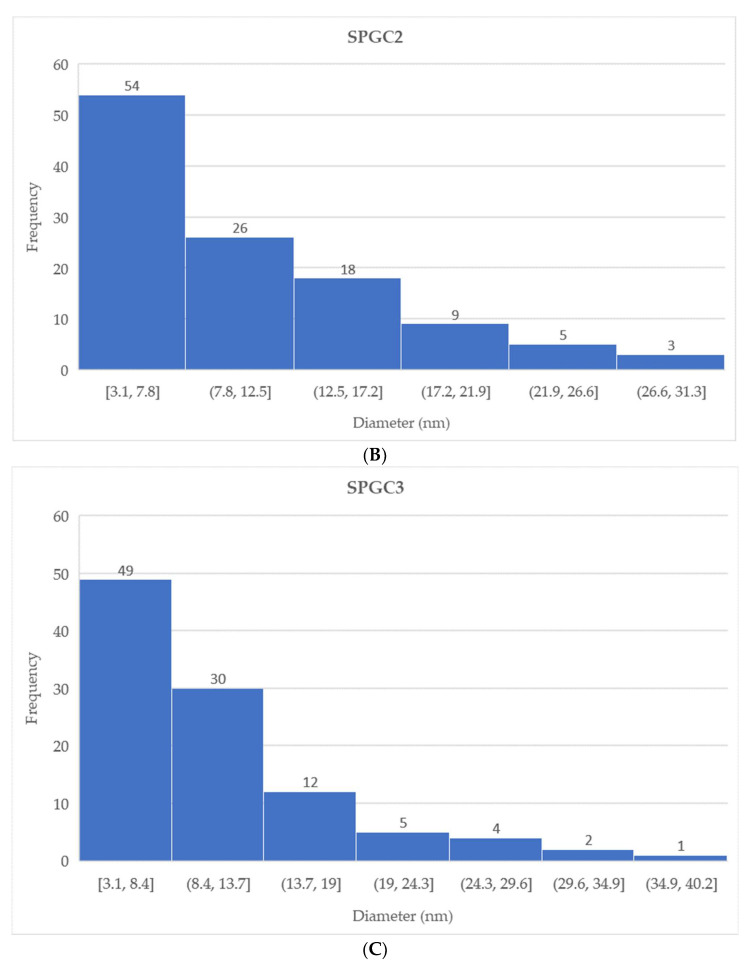

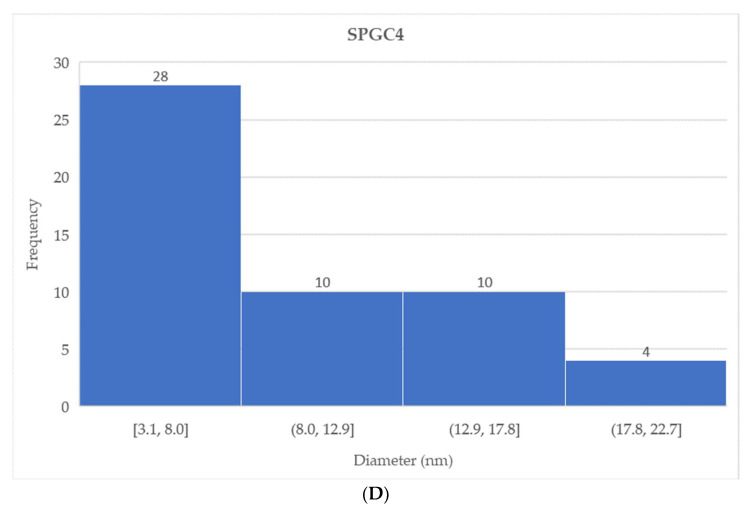
The number and size of starch granules cluster as the citric acid content of the solution increases, (**A**) 0.46 wt% CA (SPGC1), (**B**) 0.92 wt% CA (SPGC2), (**C**) 1.38 wt% CA (SPGC3), (**D**) 1.83 wt% CA (SPGC4).

As shown in Figure 8, the selected areas of the SEM images analyzed by the open-source ImageJ software package demonstrate the micro-sized clusters of starch granules and the edges of the peaks and depths on the material’s surface. Moreover, the distribution and area of starch clusters on the surface of each material (SPGC1-SPGC4) were calculated (Figure 9). The total number of cluster peaks for each sample confirms that the surface becomes smoother as each solution’s citric acid content increases. For example, the total number of peaks on the SPGC1 sample surface was recorded at 115 compared to 52 points on the surface of SPGC4.

**Figure 10:**
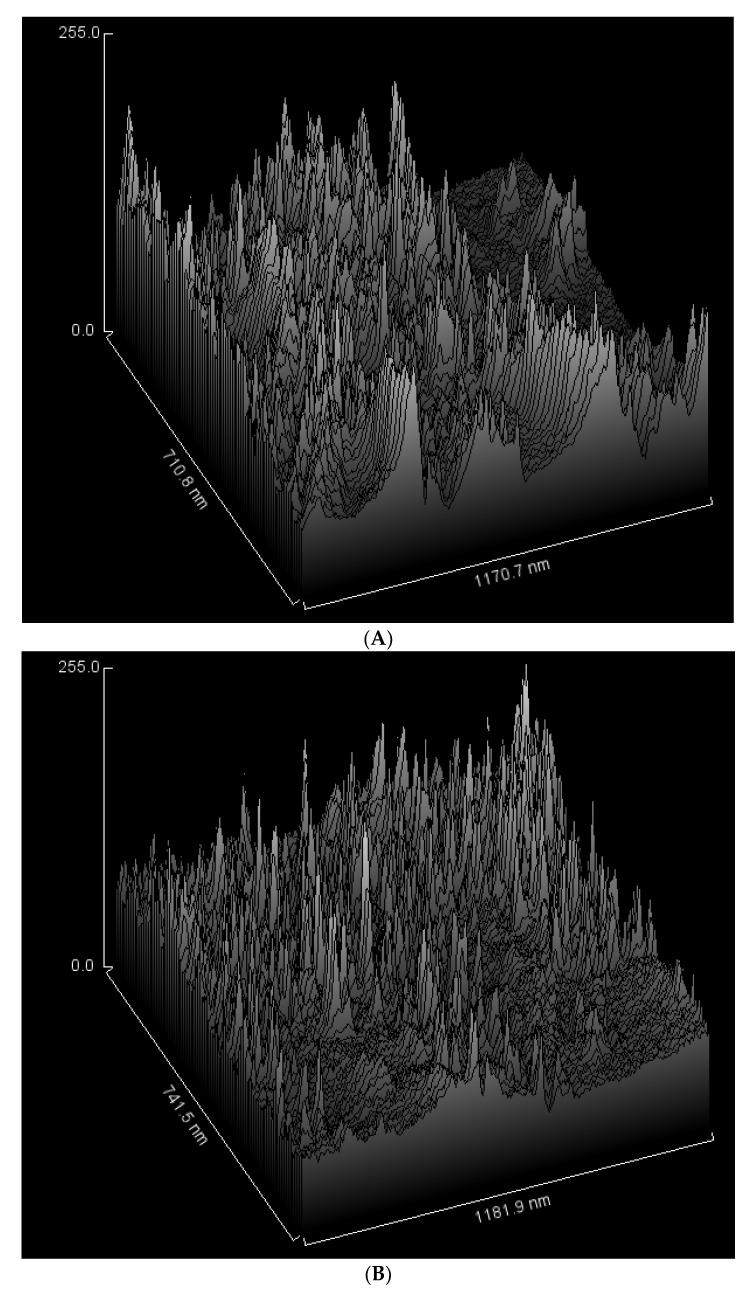

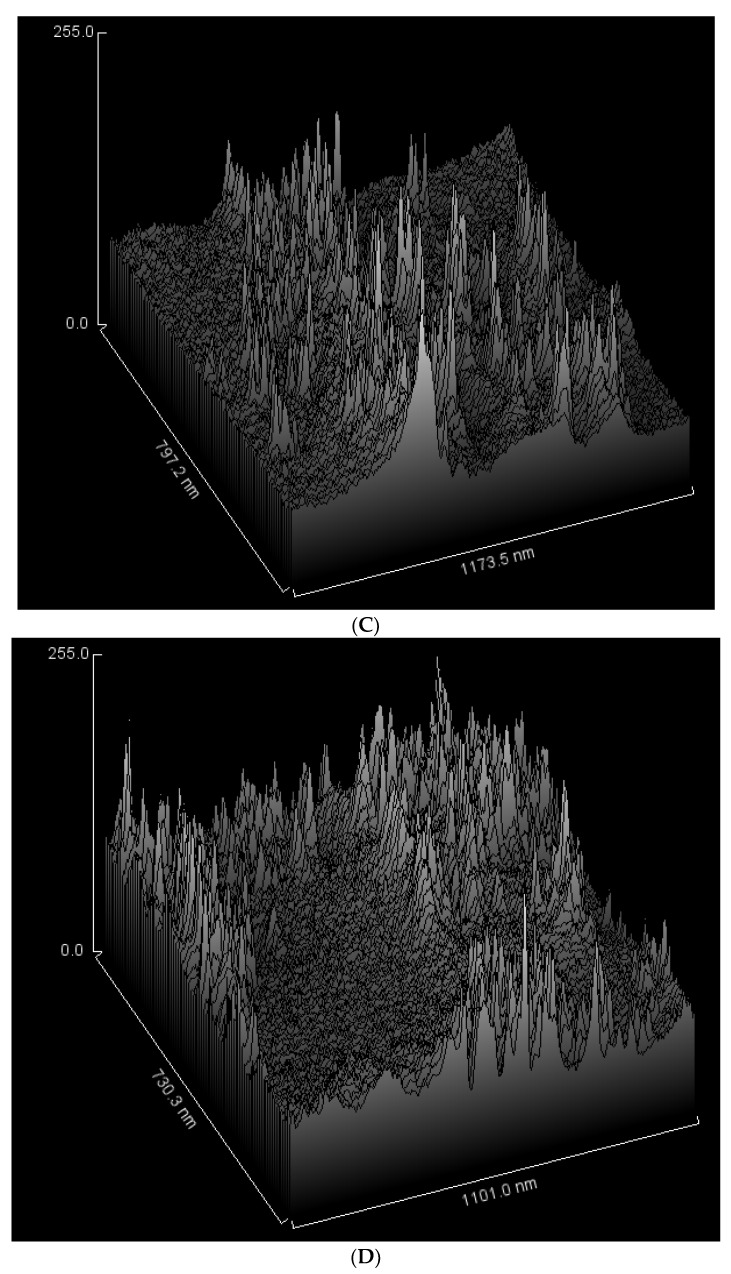
The surface roughness of the starch-based dressings as the citric acid content of the solution increases, (**A**) 0.46 wt% CA (SPGC1), (**B**) 0.92 wt% CA (SPGC2), (**C**) 1.38 wt% CA (SPGC3), (**D**) 1.83 wt% CA (SPGC4).

Additionally, in order to assess the samples’ surface roughness further, the same selected areas in Figure 8 were used to generate surface plots using ImageJ software, as shown in Figure 10. It can be concluded that various sizes of clusters were created in the sample structure matrix; small ones are well embedded in the PVA and the citric acid structure, where the surface is smoother, and the rest of the area is rougher and where the broader and taller clusters were created. Those larger clusters are starch particles that have not been fully embedded in the structure matrix; therefore, they act as space fillers in the gaps between PVA and citric acid.

## 4. Discussion

In this work, a novel method was implemented to prepare the transparent starch-based material and study the characteristics of this material as a wound-dressing. This blend is composed of starch, polyvinyl alcohol, glycerol, and citric acid. Four different contents of citric acid, 0.46 to 1.83 wt%, were tested in order to confirm the integrity of the preparation process. The fabrication process started by dissolving PVA in deionized water, and, meanwhile, the starch and DI water were mixed using an ultrasonic mixer. The literature [27,28,29,32] suggests that glycerol and citric acid as plasticizers and crosslinking agents help to improve the films’ flexibility and antibacterial properties. The prepared films (SPGC1-SPGC4) had a smooth and homogeneous surface. The WVT analysis showed that these materials had the optimal water vapor transition rate to provide enough oxygen and absorb body fluids around the wound milieu. These two characteristics sufficiently promote the wound-healing process [41]. UV–Vis spectroscopy technique measured and confirmed the transmittance values for each material [42,43,44]. To evaluate the roughness and polymer structure when the citric acid concentration was increased, these wound dressings’ surface morphology and structure were examined using SEM imaging and the ImageJ processing software; the results confirmed that the materials’ surface gets smoother by increasing the CA percentage content. In addition, the degradation-related physical characteristics of the starch-based/PVA films are similar to those described in publications on wound dressings [15,27,28,29]. The starch films demonstrated effective antibacterial inhibition zones against *E. coli* and *S. aureus*. pH measurements were taken, and it was determined that the growth of bacteria could be halted within the wound area [39,40]. Future research on this topic could look at monitoring the wound-healing steps in humans and animals after treatment with this type of protective material, modifying the composition of the dressing solution, and developing an optimization model to increase and personalize the performance of these wound dressings for each patient.

## 5. Conclusions

In this paper, transparent starch-based/polyvinyl alcohol/citric acid/glycerol material was prepared by the modified casting method. Then, the new solution’s water vapor transmission (WVT) was established and evaluated through the existing literature. The experiment outcomes demonstrated that the necessary WVT around the wound milieu may well be sufficiently supplied. The starch-based wound-dressing tests showed good dispersion and homogeneity by SEM imaging and ImageJ software surface plot analysis. Additionally, the UV–Vis spectroscopy method demonstrated the UV light transmission of the polymer composite network. This study resulted in a developed wound-dressing film with suitable characteristics, such as exceptional mechanical properties, transparency, drug delivery, antibacterial properties, and a moist environment around the wound region, to invigorate the best possible wound-healing techniques.

## Figures and Tables

**Table 1:** The composition of films.

Sample	ST: PVA: GLY (wt%)	CA (wt%)
SPGC1	2.3: 2.3: 1.8	0.46
SPGC2	2.3: 2.3: 1.8	0.92
SPGC3	2.3: 2.3: 1.8	1.38
SPGC4	2.3: 2.3: 1.8	1.83

**Table 2:** Young’s moduli of the samples.

Sample	Young’s Modulus (MPa)
SPGC1	29.2 ± 1.7
SPGC2	28.4 ± 1.6
SPGC3	26.5 ± 1.4
SPGC4	25.7 ± 1.05

## Data Availability

All the results related to the results published in this paper are presented within the article.

## Supplementary Materials

The following supporting information can be downloaded at: https://www.mdpi.com/article/10.3390/mi13060960/s1, Figure S1: Starch-based wound-dressing preparation steps, Figure S2: The average thickness of the samples (mm).
