# Supplementary material for: Preparation and Characterization of Eco-Friendly Transparent Antibacterial Starch/Polyvinyl Alcohol Materials for Use as Wound-Dressing
Source: Micromachines (Basel). 2022 Jun 17;13(6):960. doi: 10.3390/mi13060960 (PMC9231090; doi:10.3390/mi13060960)
Supplement: Supplementary file 1 [file micromachines-13-00960-s001.zip › micromachines-1756474-supplementary.pdf]

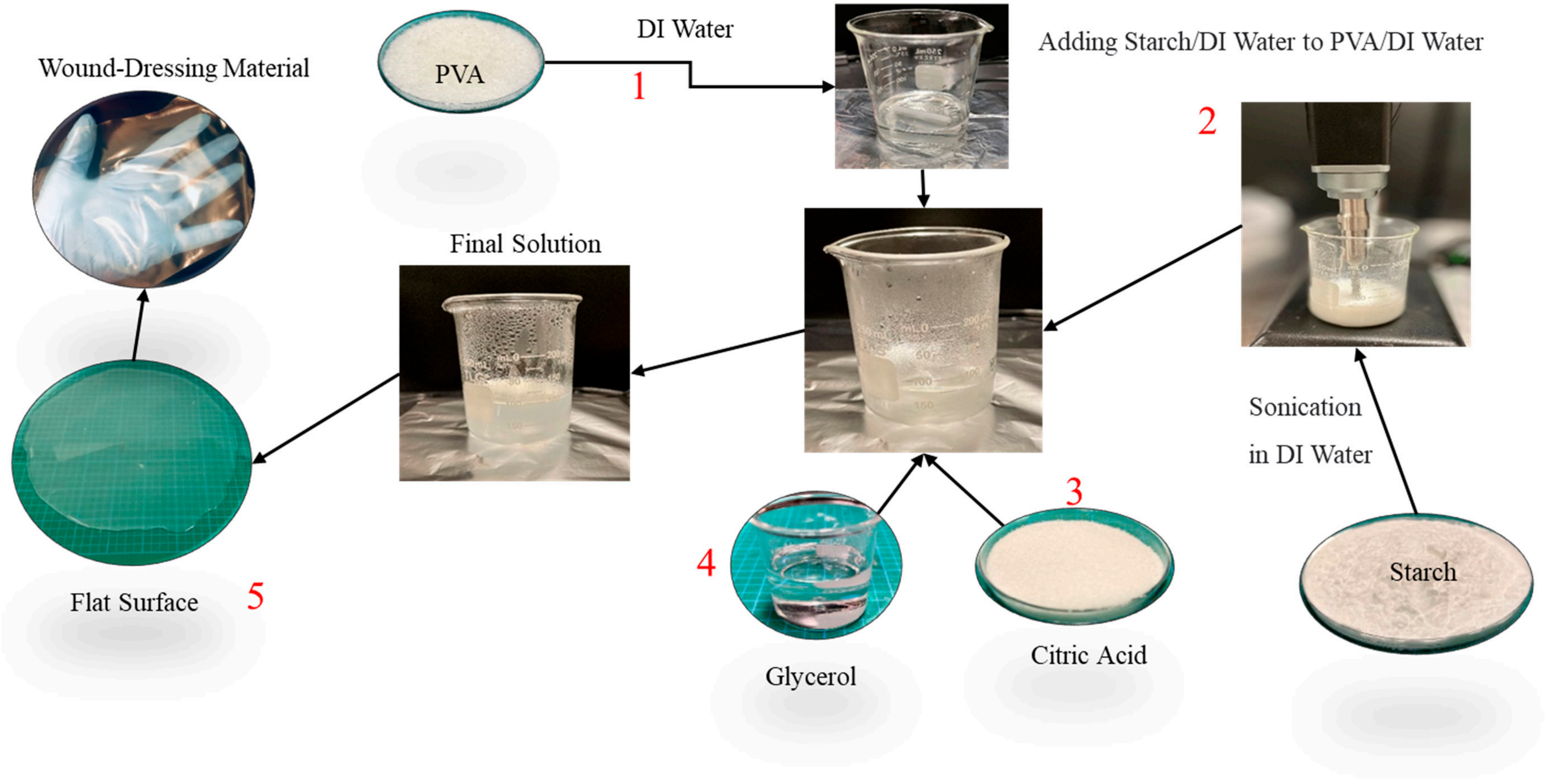

Figure S1. Starch-based wound dressing preparation steps.

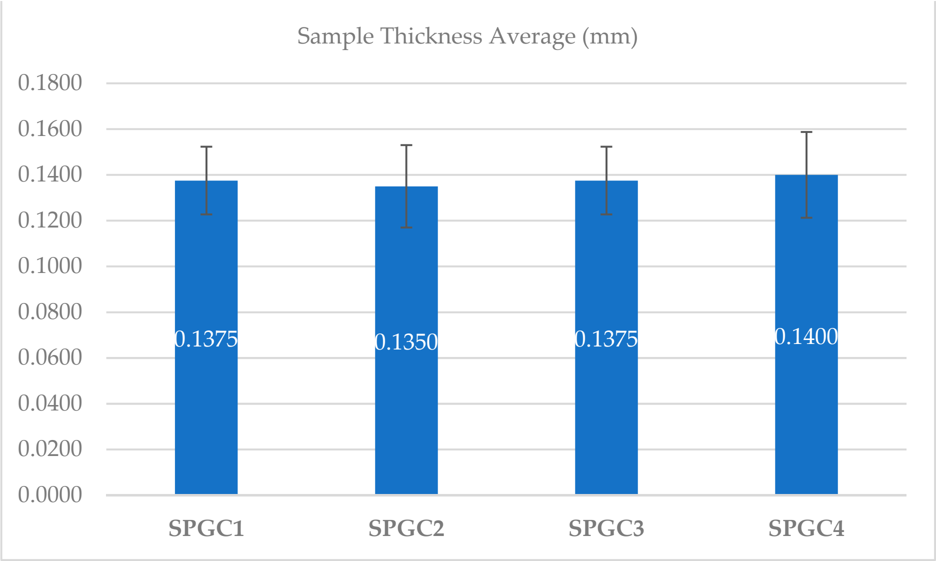

Figure S2. The average thickness of the samples (mm).
